# QUEST: Eliminating Online Supervised Learning for Efficient Classification Algorithms

**DOI:** 10.3390/s16101629

**Published:** 2016-10-01

**Authors:** Ardjan Zwartjes, Paul J. M. Havinga, Gerard J. M. Smit, Johann L. Hurink

**Affiliations:** Faculty of Electrical Engineering, Mathematics and Computer Science, University of Twente, Enschede 7500AE, The Netherlands; p.j.m.havinga@utwente.nl (P.J.M.H.); g.j.m.smit@utwente.nl (G.J.M.S.); j.l.hurink@utwente.nl (J.L.H.)

**Keywords:** wireless sensor networks, unsupervised learning, classification algorithms, Naive Bayes, semi-supervised learning, adaptive

## Abstract

In this work, we introduce QUEST (QUantile Estimation after Supervised Training), an adaptive classification algorithm for Wireless Sensor Networks (WSNs) that eliminates the necessity for online supervised learning. Online processing is important for many sensor network applications. Transmitting raw sensor data puts high demands on the battery, reducing network life time. By merely transmitting partial results or classifications based on the sampled data, the amount of traffic on the network can be significantly reduced. Such classifications can be made by learning based algorithms using sampled data. An important issue, however, is the training phase of these learning based algorithms. Training a deployed sensor network requires a lot of communication and an impractical amount of human involvement. QUEST is a hybrid algorithm that combines supervised learning in a controlled environment with unsupervised learning on the location of deployment. Using the SITEX02 dataset, we demonstrate that the presented solution works with a performance penalty of less than 10% in 90% of the tests. Under some circumstances, it even outperforms a network of classifiers completely trained with supervised learning. As a result, the need for on-site supervised learning and communication for training is completely eliminated by our solution.

## 1. Introduction

Advancements in miniaturisation and the declined cost of hardware have made Wireless Sensor Networks (WSN) a realistic vision: networks of tiny computers can monitor environments using sensors and wireless communication [1]. An efficient implementation of a practical WSN, however, is not a trivial task. Even on small scale WSNs, the amount of data that can be sampled by the sensor nodes is considerably more than what can be sustainably transmitted using current WSN radios with current battery technology [2,3,4].

For many applications, the raw sensor data itself is not of interest. For example, in domestic fire detection [5], carbon-dioxide readings do not necessarily need to reach a human operator. The presence of a fire, however, is the important information. In applications like this, processing data locally on the deployed sensor node with the goal of extracting the relevant information, for example the presence of a fire, eliminates the need for the transmission of raw data. This significantly reduces the amount of data that needs to be transmitted. From now on, we will call this online processing [2].

Online processing comes in many forms, ranging from simple schemes to compress the data to complex event recognition algorithms that draw high-level conclusions [2]. Especially, this last group of algorithms result in considerable reductions in communication by removing the need to transmit the raw sensor readings. Taking into account that the energy needed to transmit a few bytes of data already is significant [3,4], it is clear that online processing is a promising area of research for WSNs. One important example of the application of intelligent algorithms on WSNs is the use of classification algorithms to draw conclusions from raw sensor readings, e.g., classifying the samples for a given moment in time as “fire” or “no fire”.

Processing data using online classification algorithms is a challenging task [2]. In order to get correct classifications, training data is needed first. Supervised learning can be applied in two ways: online learning and offline learning. For offline learning, the sensor nodes send their measurements to a central location where individual classifiers are trained for each sensor node. After the training phase, these classifiers are deployed on the sensor nodes.

Online supervised learning works the other way around: each node trains a classifier with its own local data. Instead of transmitting the data, the desired classification outputs or labels are sent to all of the sensor nodes. Each sensor node uses these outputs to update their own local classifier.

Considering that wireless communication requires a lot of energy [4], the initial training process can deplete a significant part of the available energy of the sensor nodes. For example, if we were to apply online learning on a sensor network of *n* sensor nodes over a period of *t* seconds with a sample rate of *f* Hz, the nodes would have to receive a total of tfn messages containing the desired classification output on their location. To train the sensor network for the dataset we use in this paper [6], for a period of one week, the nodes have to receive a total of over 13 million messages under the assumption that the network topology allows all nodes to directly receive the messages without intermediate hops. More complex network topologies could require a multitude of this number of messages.

Training data needs to be representative for the relevant situations of what the classifier might encounter, which complicates training [7]. For example, if sensor readings for a classification problem are influenced by environmental conditions, proper online training might require a network to run in training mode for a year in order to account for all seasonal effects. Another drawback of supervised learning is the possible need for human interaction. If a human needs to train the network by labelling the desired outputs, this requires expensive man-hours. Furthermore, due to variations in hardware and deployment locations, this complicated process needs to be repeated every time, and a sensor node needs to be replaced or installed in a new environment. As a consequence, the maintenance of a WSN using a classification algorithm for a long period of time is often highly impractical.

The use of classification algorithms on WSNs is not a new idea. For example, Ref. [8] describes the detection of environmental events using Bayesian classifiers on WSNs. The work described in [9] describes a scalable method for collaborative event detection on WSNs. Fault tolerant classification is the subject of [10]. The feasibility of sound classification on WSNs is the subject of [11]. All these papers have in common that they propose classification algorithms or applications for classification algorithms in the scope of WSNs.

Another application related to WSNs is the field of Body Area Networks (BAN). BANs are small networks of devices on or around a human body. Applications for BANs include gait analysis for rehabilitation purposes [12,13], activity monitoring [14] and athlete coaching [15]. For these applications, online classification is frequently applied. Ref. [12], for example, applies a distributed Hidden Markov Model to detect the gait phases of a person walking. BANs have many similarities with WSNs, and could even be defined as small WSNs. A BAN is a network of small battery powered devices with sensors to observe an environment. However, there are some key differences to WSNs in general. A BAN is usually deployed in a very accessible area: around a human body. This means that battery replacement or recharging is not a major challenge. Furthermore, communication distance is limited to several meters, making communication more energy efficient. As such, the techniques suitable for a BAN are not necessarily applicable for WSNs.

Looking at the used classification approaches, we can state that many different approaches have been proposed over the years. Our work is based on the Naive Bayes classifier [16], a well-known classification technique that has been used for sensor networks in other research (see e.g., [5,17,18,19]).

In this work, we introduce QUEST, which stands for QUantile Estimation after Supervised Training. QUEST is a versatile classification algorithm for WSNs. QUEST aims to combine some advantages of unsupervised learning with the accuracy of supervised learning. Advantages of unsupervised learning algorithms include that they need no human involvement, which saves valuable man-hours [7]. Another advantage is the fact that there is no dependency on wireless communication, as learning is done based on local samples.

The mixture of supervised and unsupervised learning by itself also is not a new idea. For example, in [20], it is applied for time series forecasting. Note that, for many applications, the clustering of data using unsupervised learning does not provide meaningful information without supervised labeling of the clusters. The contribution of this work is a scheme where unsupervised learning is not only used to create clusters that are used in an supervised learning settings, as in [17], but the continuation of unsupervised learning in a deployed sensor node to adapt an existing classifier to the particular environment of the deployed sensor node.

The division of an input space into areas of interest using unsupervised learning is one of the key principles used in this paper. Some examples of the many algorithms in this area are Kohonen networks [21], which are self organising feature maps, and the $P^{2}$ algorithm (see [22,23]), which is a heuristic to estimate quantiles. However, there are numerous other published approaches (see e.g., [24,25]).

The remainder of this paper is organised as follows. Section 2 describes the principles behind QUEST and the experiments used to show the efficiency of QUEST, and Section 3 gives the results of these experiments. The conclusions of this work are presented in Section 4.

## 2. Method

We have restructured the Method section In this section, we describe our proposed solution and the experiments performed to verify its efficiency. The working of the QUEST algorithm is described in Section 2.1, Section 2.2 describes the experiments performed on a real-life dataset and Section 2.3 describes the experiments performed using simulated data.

### 2.1. Proposed Solution

This part was moved into the method section In this paper we introduce QUEST, a classification algorithm that combines supervised learning with unsupervised learning in a way that enables efficient deployment of a classification algorithm on a WSN. QUEST is based on the principles behind the Naive Bayes classification [16].

**Figure 1:**
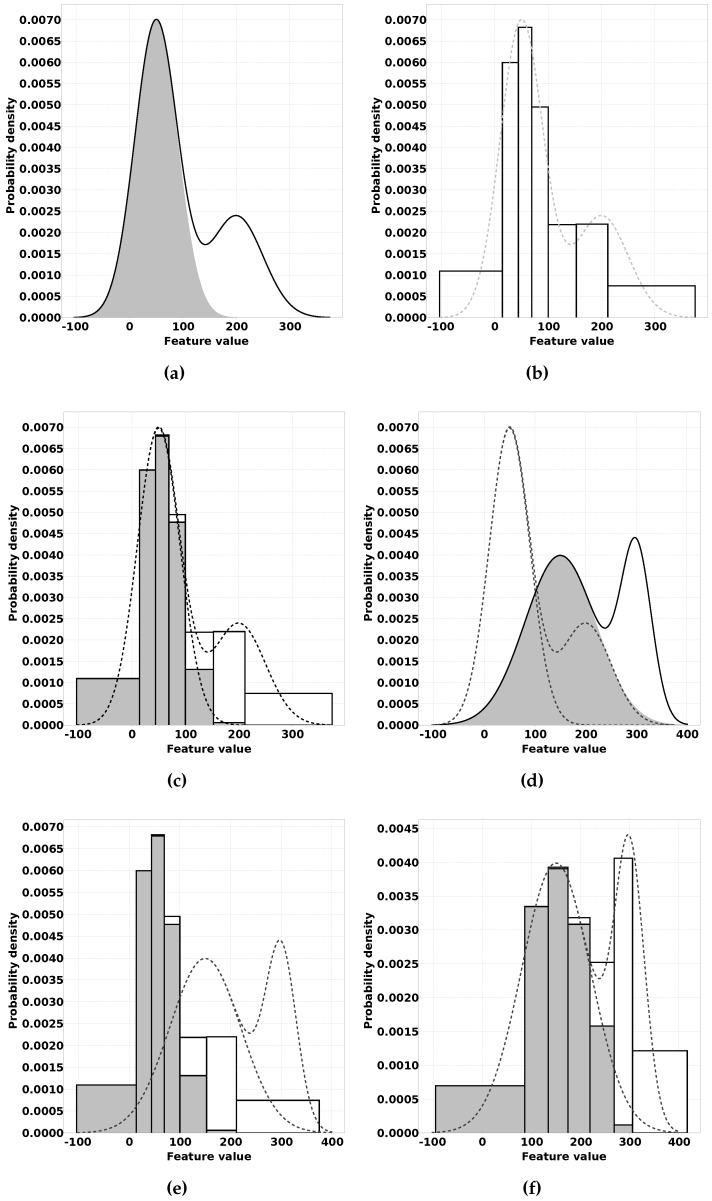
Visualisation of the offline phase of QUEST (QUantile Estimation after Supervised Training). Gray and white areas represent different desired output classes. (**a**) Example distribution; (**b**) The *x*-axis is divided in intervals using an unsupervised learning heuristic. The borders lie on $i_{1}\ldots i_{6}$, $i_{n}$ is the $\frac{1}{n}$ quantile; (**c**) The distribution of classes, is empirically estimated for each quantile; (**d**) A different data distribution encountered by a sensor node running QUEST, the original distribution is shown as dashed lines; (**e**) Initially, the distribution of the quantiles for the classifier does not match the new distribution; (**f**) QUEST applies an unsupervised learning heuristic to update the quantiles for the modified distribution.

In the following section, we clarify our proposed solution using a simulated classification problem. The inputs are generated by drawing samples from a distribution consisting of two Gaussian distributions representing two possible classification outputs: $c_{1}$ and $c_{2}$. An example of such a distribution is shown in Figure 1a. This figure is a stacked area chart that shows a probability density function, where the gray area represents $c_{1}$ and the white area represents $c_{2}$.

Naive Bayes classifiers process input values, which are raw sensor measurements or features derived from those measurements, and automatically assign a classification, or label, to those input values. An example of a derived feature can be a frequency component derived from a Fast Fourier Transform of the output of a vibration sensor. An example of the output of the Naive Bayes classifier can be fire or no fire, or “vehicle present in the detection range”.

To create an output, Naive Bayes classifiers use Bayesian statistics and Bayes’ theorem to find the probability P(c|E) that a given observation $E=(x\left(s_{1},t\right),x\left(s_{2},t\right),...,x\left(s_{n},t\right))$ belongs to a class *c*, where $x(s_{i},t)$ is the value of feature $s_{i}$ at time *t*. This probability P(c|E) is estimated using Equation (1) [16]:

(1) $$P\left(c|E\right)=\frac{P(E|c)P(c)}{P(E)}.$$

In order to make a classification decision, the Naive Bayes algorithm calculates P(c|E) for each c∈C. The class with the highest probability is the final classification.

In this case, class *c* can, for example, be the class of samples where there is a fire, $x(s_{i},t)$ is the output of sensor $s_{i}$ on time *t*. The algorithm is called naive because of the assumption that all the inputs $x(s_{i},t)$ have an independent contribution to P(c|E).

Accurately estimating the probabilities for Equation (1) is important for the classification result. One approach is to model the inputs using standard data distributions, such as the Gaussian distribution [7]. Another approach is to divide the input space into separate parts for which the probabilities are estimated based on supervised learning. For each part of the input space, the fraction of samples belonging to each class is determined empirically. These fractions are used to determine the probability that an input will fall in a certain interval given a classification *c*.

In [17], we already presented an approach to efficiently train a Naive Bayes classifier for WSN applications. This approach works in two steps. The first step is to divide the input space for each feature using unsupervised learning. The second step is to empirically estimate the distribution of each class of observations over these intervals. These distributions are what the Naive Bayes classifier uses to determine the classification output. The result of the second step for the example distribution is shown in Figure 1c. Once again, in this figure, the gray areas represent output $c_{1}$ and the white areas represent output $c_{2}$. Algorithm 1 gives a description of this process.

QUEST is developed with [17] as a starting point. The first improvement came through the observation that where unsupervised learning provided a functional division of the input space for the Naive Bayes classifier, the actual goal was to divide the input space by estimated quantiles so that each interval between two neighbouring quantiles has the same probability density. The reason for this is that with equally populated intervals, the chance that an estimated probability for a class in an interval has a large error compared to the actual probability becomes smaller. For this, the *p*-quantile is used. It is defined as the value below which 100p percent of the distribution lies (e.g., the median is the 0.5 quantile). In our example figures, we use a division of the input space in seven intervals for which the borders lie on $i_{1}\ldots i_{6}$, where $i_{n}$ is the $\frac{1}{n}$ quantile (see Figure 1b). Therefore, for QUEST, we have selected an unsupervised learning algorithm that aims to learn the correct quantile estimations.

Unsupervised quantile estimation can be done in several ways. The approach used in [17], which is a modified version of the Kohonen network algorithm [21], gives estimates roughly correlated with the quantiles. Other options are the regular Kohonen network algorithm or the $P^{2}$ algorithm [23].

With QUEST, we solve a problem that was left unsolved by [17]. Where the approach of [17] allows for memory efficient training of Naive Bayes classifiers, online or offline supervised training still requires a lot communication. In this paper, we propose a solution where unsupervised learning is not only applied before supervised learning, but also after supervised learning to adapt a trained classifier. In the first phase, which we call the offline phase, a single classifier is trained using the described process from [17] but with an improved quantile estimation algorithm. The data this classifier is trained with should be carefully selected to be a good representation for the complete classification problem. This data can e.g. be generated in lab conditions where various conditions that can be encountered are created. Figure 1a–c show this process on an example distribution created by two Gaussian distributions, one for each class. The results of the two steps of this algorithm are the knowledge needed to implement a Naive Bayes classifier trained for the example data. The classifier resulting from this data will be pre-loaded on all sensor nodes.

**Algorithm 1:** Naive Bayes based on unsupervised clustering  $C:=\{c_{1},\ldots ,c_{n}\}$, possible classification outputs  $d_{t}:=$ desired classification output at time *t*  $o_{t}:=$ actual classification output at time *t*  $x(s_{n},t)$ the value of feature $s_{n}$ at time *t*  $E_{t}:=\left\{x\left(s_{1},t\right),\ldots ,x\left(s_{n},t\right)\right\}$, observation at time *t*  $P_{s}\left(x\right):=$ the interval number sample *x* lies in for feature *s*  $\hat{P}\left(i,s_{n},c_{n}\right):=$ the estimated probability of $P(s_{n})=i$ given $d_{t}=c_{n}$  $\hat{P}\left(c_{n}\right):=$ the estimated overall probability of class $c_{n}$  t:=1  ▹
Unsupervised phase  **while**
*¬Unsupervised phase finished*
**do**    **for**
*$s:=s_{1}$*
**to**
*$s_{n}$*
**do**      update $P_{s}$ with sample x(s,t) using unsupervised learning    **end**    t:=t+1  **end**  ▹
Supervised phase  **while**
*¬Supervised phase finished*
**do**    **for**
*$s:=s_{1}$*
**to**
*$s_{n}$*
**do**      update the statistics used to determine $\hat{P}\left(i,s,c_{n}\right)$ given $d_{t}$ and $P_{s}\left(x\left(s,t\right)\right)$      update the statistics used to determine $\hat{P}\left(c_{n}\right)$ given $d_{t}$    **end**    t:=t+1  **end**  ▹
Classification phase  **while**
*¬Classification phase finished*
**do**    ▹
See Equation (1)    $\hat{P}\left(c_{n}\right):=\hat{P}\left(c_{n}\right)\prod_{s=s_{1}}^{s_{n}}\frac{\hat{P}\left(P_{s}\left(x\left(s,t\right)\right),s,c_{n}\right)}{\hat{P}\left(P_{s}\left(x\left(s,t\right)\right)\right)}$    $o_{t}:=c\in C|\left(\neg \exists c^{*}|\hat{P}\left(c^{*}\right)>\hat{P}\left(c\right)\right)$    t:=t+1  **end**

In the second phase, which we call the online phase, each node on a deployed network uses the unsupervised learning heuristic from step one to update the quantiles and thereby the division of the input space. Next to that, the classifier contributes to the classification output of the overall network. We expect that, at first, the network will not perform very well, since each node uses a classifier that was trained for a sensor node deployed under different circumstances. These different circumstances may have influence on the data distribution. For example, variations in temperature, distance to the observed phenomenon, hardware, etc. may have a negative impact on the classification performance. An example of a possible modified distribution is shown in Figure 1d, where the original distribution is shown as dashed lines and the new distribution of the classes is shown as gray and white areas stacked on top of each other. The pre-loaded data for the classifier trained in the offline phase in this case does not match the data distribution for the new circumstances (see Figure 1e).

To handle these variations, each node continues with the unsupervised learning. This leads to updates to the quantile estimations to match the sensor readings for the location on which the sensor node is deployed. By providing unlabelled samples, the intervals will gradually change to reflect the new conditions, resulting in the data shown in Figure 1f.

**Figure 2:**
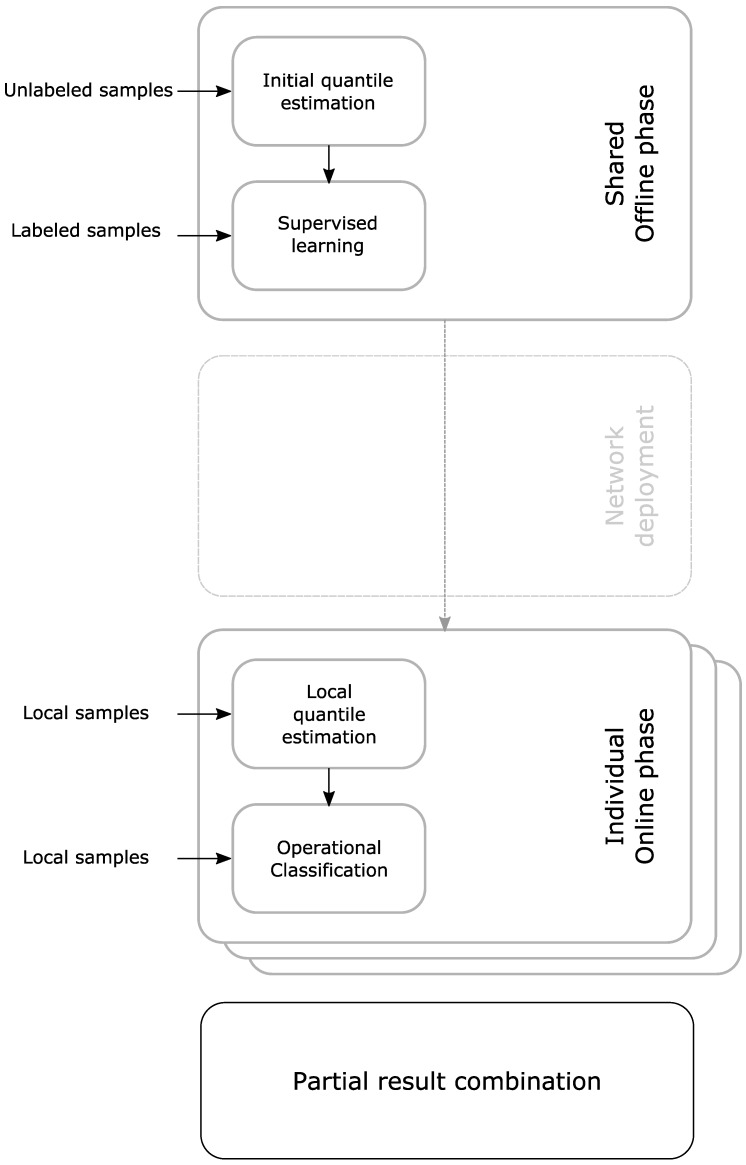
Block diagram of the phases of QUEST.

Figure 2 gives an overview of the phases of QUEST and Algorithm 2 shows the working of QUEST in pseudocode. In the remainder of this section and Section 3, we describe experiments and results to give more details about the way QUEST works.

**Algorithm 2:** QUEST (QUantile Estimation after Supervised Training) updates the unsupervised clustering of the feature space after deployment  t:=1  ▹
Offline unsupervised training phase  **while**
*¬Unsupervised phase finished*
**do**    **for**
$s:=s_{1}$
**to**
$s_{n}$
**do**      update $P_{s}$ with sample x(s,t) using unsupervised learning    **end**    t:=t+1  **end**  ▹
Offline Supervised training phase  **while**
*¬Supervised phase finished*
**do**    **for**
$s:=s_{1}$
**to**
$s_{n}$
**do**      update the statistics used to determine $\hat{P}\left(i,s,c_{n}\right)$ given $d_{t}$ and $P_{s}\left(x\left(s,t\right)\right)$      update the statistics used to determine $\hat{P}\left(c_{n}\right)$ given $d_{t}$    **end**    t:=t+1  **end**  ▹
End of offline phase, each node is now deployed with the trained classifier  ▹
Online unsupervised training phase  **while**
*¬Online unsupervised phase finished*
**do**    **for**
$s:=s_{1}$
**to**
$s_{n}$
**do**      update local $P_{s}$ with sample x(s,t) using unsupervised learning    **end**    t:=t+1  **end**  ▹
Classification phase  **while**
*¬Classification phase finished*
**do**    $\hat{P}\left(c_{n}\right):=\hat{P}\left(c_{n}\right)\prod_{s=s_{1}}^{s_{n}}\frac{\hat{P}\left(P_{s}\left(x\left(s,t\right)\right),s,c_{n}\right)}{\hat{P}\left(P_{s}\left(x\left(s,t\right)\right)\right)}$▹
see Equation (1)    $o_{t}:=c\in C|\left(\neg \exists c^{*}|\hat{P}\left(c^{*}\right)>\hat{P}\left(c\right)\right)$    t:=t+1  **end**

### 2.2. Experiments on Real Data

#### 2.2.1. Real-Life Dataset

For experiments using a real-life dataset, we used the SITEX02 dataset [6]. The SITEX02 dataset is a dataset that was fully made available online, which is fairly uncommon. As such, it is an excellent foundation for WSN experiments. This dataset provides seismic and acoustic sensor readings for experiments with different types of vehicles that drove through an area where a network was deployed. The sensor network was Sensoria Corporations WINS NG 2.0 build using 22 WINS NG sensor nodes (Version 2.0, Sensoria Corporation, San Diego, CA, USA) [26]. The size of the target area is approximately 900 × 300 m^2^. In this dataset, ground truth location information on the vehicles is provided based on GPS trackers placed on the vehicles. The exact details of the sensors were not provided in [6] but are of limited importance for this work.

Two different type of vehicles were used in this dataset: the Dragon Wagon (DW) and the Assault Amphibian Vehicle (AAV). The dataset contains nine runs for the AAV vehicle through the target area and 11 runs for the DW vehicle. The seismic and acoustic sensor readings are both processed into features using Fast Fourier Transforms. Each sample stream was divided into 50 frequency bands for which the intensity was recorded once each second. The result is a dataset with 100 features, each with a sample rate of 1 Hz.

For this paper, we assigned the samples to one of the following classes: (1) DW within the detection radius; (2) AAV within the detection radius; and (3) no vehicle within the detection radius. For the detection radius, we have experimented with several values, namely 50 m, 75 m, 100 m and 125 m. These values influence the training of the classifier in two ways. First, a larger radius means that a node has to detect a vehicle over a longer distance, which is more complicated since acoustic and seismic signals fade over distance, resulting in a lower signal-to-noise ratio. On the other hand, a larger detection radius means that the dataset contains more samples with a vehicle in the detection radius, which means that the classification can be based on more information.

**Figure 3:**
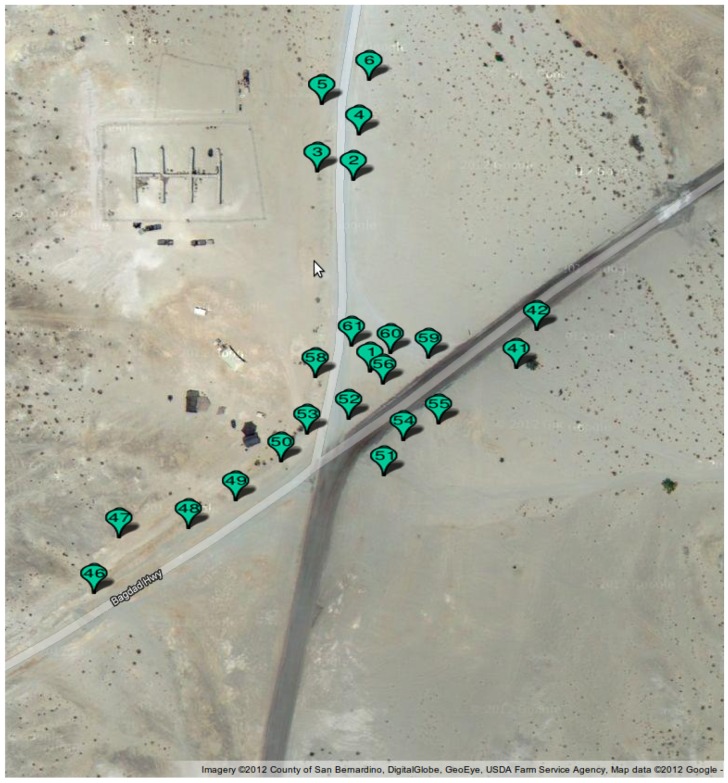
Google maps view of the SITEX02 network deployment.

**Figure 4:**
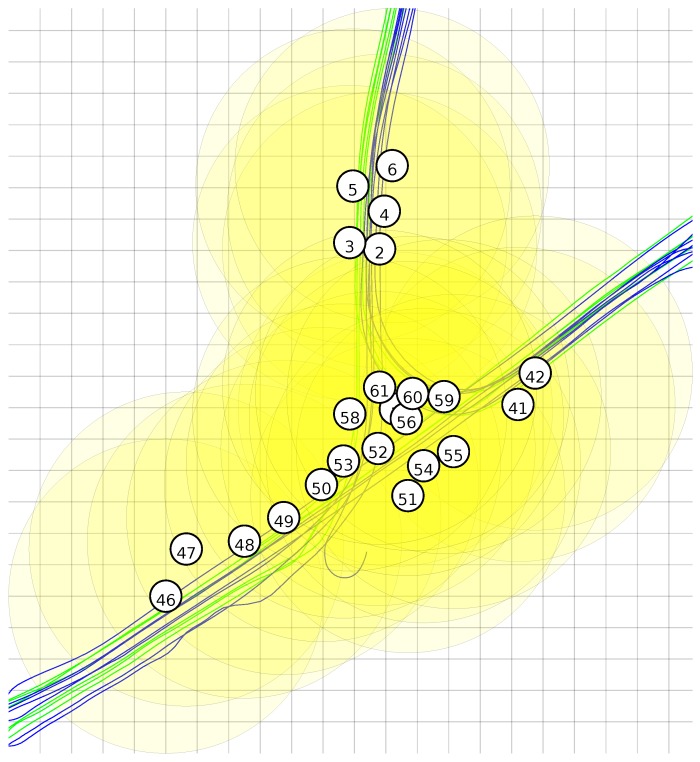
Detection areas for a radius of 100 m and vehicle traces; grid size is 20 m.

To label all the samples in the dataset, we used the recorded vehicle positions and the given node coordinates. The deployment of the nodes of the network can be seen in Figure 3. Figure 4 shows the node locations, vehicle traces and the detection range. It can be seen that a lot of overlap is present.

#### 2.2.2. Fusion Model

A challenging aspect of the SITEX02 dataset is that the events are not global. If a vehicle is in the detection range of one node, it is not necessarily in the detection range of another node. Regular Naive Bayes classification fuses all input probabilities using multiplication. This approach is not suitable to combine the classification outputs of multiple nodes from the SITEX02 dataset. When a single node correctly determines that the probability of a vehicle being in its detection radius is zero, other nodes still need to be able to detect the presence of the vehicle. A multiplication by zero would prevent that.

In order to take the relation between detection areas into account, we created a fusion model. In this paper, we have chosen to use a simple model based on the distance between sensor nodes for this paper. In the selection of this approach, we did not try to achieve the best classification result possible, but merely aimed for a reasonable solution that allowed us to demonstrate the performance impact of QUEST compared to regular Naive Bayes.

For the fusion method, we have chosen a weighted sum where the influence of each node was determined using Equation (2). In this equation, $S_{f}\left(n,C,t\right)$ is the fused classification score for node *n* and classification class *C*. For example, S(i,C,t) can represent the local score of detecting a Dragon Wagon at time *t* for node *i*. The weight w(n,i) of the contribution of node *i* for the result of node *n* is determined based on the distance d(n,i) between the nodes *n* and *i* and the detection radius *r*:

(2) $$\begin{array}{ccc} S_{f}\left(n,C,t\right) & = & \sum_{i}w\left(n,i\right)S\left(i,C,t\right),\\ w(n,i) & = & \left\{\begin{array}{cc} 0 & d(n,i)>r,\\ \frac{r-d(n,i)}{r} & d(n,i)\leq r. \end{array}\right. \end{array}$$

#### 2.2.3. Feasibility Study Based on Correlation

QUEST is based on the assumption that the probability distribution over the intervals determined for a single node is related to the distribution determined for all other nodes. For QUEST, we adapt quantile estimations in the online phase, the probability distribution of the classes over the intervals is not changed during the online phase. If that assumption is correct, we would expect to observe a high correlation between the class probabilities in corresponding intervals between classifiers for different sensor nodes that were trained using supervised learning.

We have verified the validity of this assumption by training classifiers for each sensor node of the SITEX02 dataset separately, using quantile estimation and supervised training. For these classifiers, we compared the probability distribution for each interval for each feature with the classifiers trained for other nodes. For each sensor in the dataset, we have determined the correlation between these values. The results of this experiment are shown in Section 3.1.1.

#### 2.2.4. Performance Compared to Regular Naive Bayes

In order to further demonstrate the validity of our approach, in a WSN application, we performed simulations to determine the accuracy of QUEST compared to regular Naive Bayes. We simulated a scenario where the nodes collaborate to determine whether the vehicles in the SITEX02 dataset are in the detection range of each individual node. This means that, for each instant for which there are measurements, each node makes a classification if there is a vehicle in its range, based on its own measurements and information given by the other nodes.

##### Baseline

Using the described fusion method, we compare the performance of our approach to the performance of a network completely trained with supervised learning. The performance of the network trained completely with supervised learning is called the baseline in the remainder of this paper. In this way, the baseline performance gives a measure of the accuracy that can be achieved using Naive Bayes classifiers on the given classification problem.

The baseline was trained using a randomly selected training set consisting of 10% of the available data. Using these trained classifiers and our fusion model, we assessed the performance of the classifiers using the distance to the Receiver Operating Characteristics (ROC) center line [27,28,29,30]. The distance to the ROC center line is a metric that gives a good indication of how well a classifier can discriminate between two classes regardless of a possible bias between the classes. This metric is preferable to, for example, accuracy, since, for heavily biased classification problems, a high accuracy can be reached without actually having a meaningful classifier. For example, when 99% of the samples are of class A, an accuracy of 99% can be achieved by always classifying samples as class A. We also used this method in previous work [19].

##### Experiment

To compare our approach to the baseline, we conducted 22 experiments, one for each sensor node from the dataset. In each experiment, we used supervised learning to train a classifier for a single node, with a dataset of 10% of the available data for that node. Next, we used unsupervised learning to adapt 21 copies of this classifier. For each copy, we used a training set with 10% of the data from another single sensor node from the dataset. Finally, we compared the performance of the 21 classifiers for each experiment to the performance of the classifier for the baseline. We calculated the relative change in the distance to the ROC center line for each classifier. By averaging the result of the classifiers for each experiment, we demonstrate the relative performance to the baseline for each of the 22 experiments.

**Figure 5:**
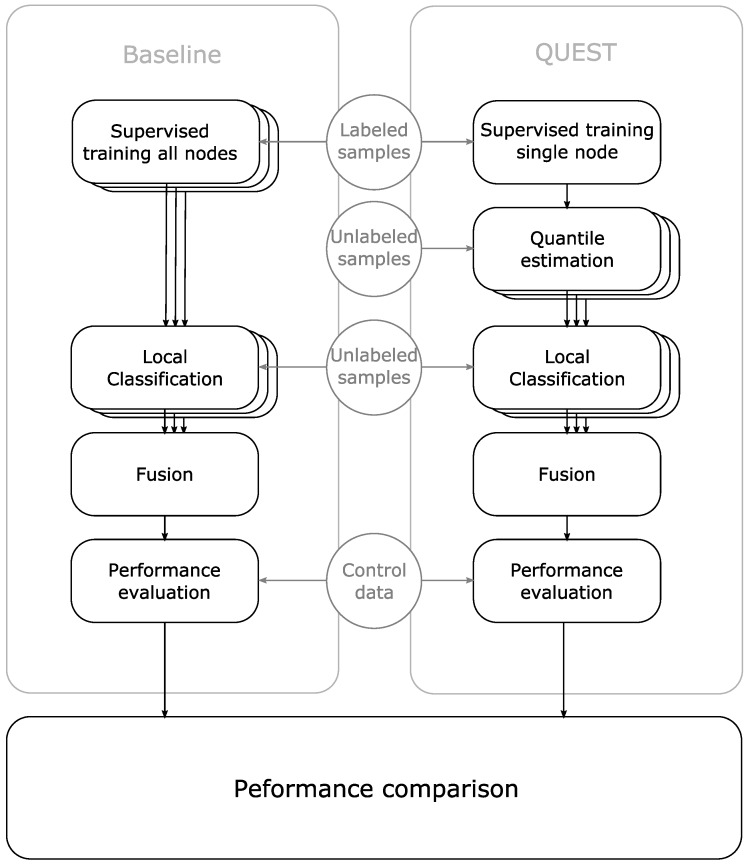
Block diagram of the procedure to compare the performance of QUEST to complete supervised learning. The QUEST classifiers are based on supervised training of a single node.

Figure 5 shows the procedure for one of the 22 experiments.

Furthermore, we have investigated if there is a clear relation between the location of a sensor node and how suitable it is to provide the supervised data from the offline phase. If the suitability is highly location-dependent, we expect that the best performing experiments are the result of an offline phase with sensor nodes that are in close proximity. If there is no clear relation between the performance of the experiment and the location of the sensor node and QUEST shows good performance, this is an indication that all nodes learned a similar classification strategy, which can be adapted by QUEST.

### 2.3. Experiments on Simulated Data

To be able to perform a detailed investigation of the properties of QUEST, we used simulated data. These experiments are described in this section. Simulated data has the advantage that it can be manipulated in order to conduct experiments using specific variations in parameters.

For the experiments using simulated data, we use samples drawn from a mixture of Gaussian distributions (Gaussian Mixture Model). We have conducted experiments using various numbers of features and output classes. Each sample consists of a value for each feature. Each feature value is drawn from a mixture of *n* Gaussian distributions where *n* is the number of classes. For each experiment, we simulated the offline phase using one mixture of Gaussian distributions and the online phase using a modified mixture. The modifications were based on a number of parameters that are described in this section.

#### 2.3.1. Comparing Quantile Estimation Heuristics

A key part of the QUEST algorithm is the quantile estimation heuristic. There are multiple algorithms that can perform this task. For the application on WSNs, low memory usage and low computational complexity are key aspects. Based on these factors, we have selected the $P^{2}$ algorithm [23], Kohonen networks [21] and a custom Self Organising Map (SOM) used in [17]. These three algorithms do not require the storage of a large number of samples in memory and do not require complex computations. The goal of these algorithms for QUEST is to accurately estimate the quantiles. To determine the accuracy of the quantile estimations, we have conducted experiments with a distribution with a known Cumulative Probability Density Function (CPDF). This implies that, for each estimated quantile, we know the desired CPDF value; for example, the 0.5 quantile has a desired CPDF value of 0.5. We have determined the Root Mean Square Error (RMSE) of the quantile estimations over multiple training iterations. We have repeated this experiment for multiple distributions and plotted the average RMSE over multiple unsupervised training iterations. The results of this experiment are shown in Section 3.2.1.

#### 2.3.2. Accuracy Evolution over Training Iterations

We have rephrased these sentencesIn our proposed solution, we expect the classification output of sensor nodes to have a low accuracy at the beginning of the online phase. This low accuracy would be the result of the quantile estimations not matching the data distribution in the new environment. During the online phase, a sensor node should adapt their quantile estimations, thereby improving the classification accuracy.

To verify this effect, we have performed an experiment where we trained a classifier for an initial sample distribution. After this training phase, we changed the data distribution by using different means and standard deviations for the Gaussian distributions for each feature for each class. We ran experiments over a number of iterations. For each iteration, we generated a sample to train the quantile estimators and a number of samples to estimate the accuracy of the classifier after training. We expect that the accuracy of the classifier over the iterations increases until a certain threshold is reached. Repeating these experiments multiple times to calculate average accuracy and determining the relative accuracy compared to a classifier trained with supervised training for the new distribution gives valuable insight in the performance of QUEST. The described test procedure is summarised in Algorithm 3, and the results of this experiment are shown in Section 3.2.2.

**Algorithm 3:** Procedure used for the simulation of QUEST to evaluate the accuracy evolution over training iterations  $n_{s}:=$ number of samples used for supervised learning  $n_{u}:=$ number of samples for initial unsupervised learning  $n_{a}:=$ number of samples used for accuracy determination  $t_{supervised}\left(c,d,n\right):=$ Train classifier *c* with *n* labeled samples from class distribution *d*  $t_{unsupervised}\left(c,d,n\right):=$ Train classifier *c* with *n* unlabelled samples from class distribution *d*  dist():= generate an initial random class distribution  modify(d):= generate a modified distribution from *d*  classifier():= generate an untrained classifier  clone(c):= create a copy of classifier *c*  accuracy(c,d,n):= accuracy for classifier *c* for distribution *d* over *n* samples  $a_{r,i}:=$ the accuracy of the classifier for repeat *r* after iteration *i*  **for**
r:= 1 **to**
*repeats*
**do**    $d_{1}:=dist\left(\right)$    $d_{2}:=modify\left(d_{1}\right)$    $c_{1}:=classifier\left(\right)$    $c_{1}:=t_{unsupervised}\left(c_{1},d_{1},n_{u}\right)$    $c_{1}:=t_{supervised}\left(c_{1},d_{1},n_{s}\right)$    $c_{2}:=clone\left(c_{1}\right)$    **for**
i:= 1 **to**
*iterations*
**do**      $c_{2}:=t_{unsupervised}\left(c_{2},d_{2},1\right)$      $a_{r,i}:=accuracy\left(c_{2},d_{2},n_{a}\right)$    **end**  **end**  $\mu_{i}:=\bar{a_{1,i}\ldots a_{n_{r},i}}$  $\left\{\mu_{1},\ldots ,\mu_{n_{i}}\right\}$
▹
Result

#### 2.3.3. Impact of the Increased Overlap Between Classes

There are multiple ways in which the modified distribution can differ from the original distribution. One of the factors that can differ is the overlap between the class distribution. When there is more overlap between classes, the classification problem becomes more complex since it is harder to distinguish between the classes. We expect that the absolute accuracy of QUEST will drop with larger overlap, but that the drop in accuracy is comparable to classifiers using fully supervised training.

In order to demonstrate this effect, we have performed multiple experiments where we limited the distance between the means of the Gaussian distributions for the various classes. A larger distance between two classes means less overlap. We repeated each experiment for each distance a number of times and calculated the mean accuracy (both absolute and relative) compared to classifiers that were trained using supervised learning. As the unit of distance between classes we used the largest standard deviation of the two distributions between which the distance is measured. This metric was chosen since the amount of overlap between two Gaussian distributions has a high correlation with this metric, and this metric easily can be changed between experiments. The procedure for this experiment is summarised in Algorithm 4, and the results of this experiment are shown in Section 3.2.3.

**Algorithm 4:** Procedure used for the simulation of QUEST to evaluate the effect of increasing the distance between class distributions  dist(δ):= generate an initial random class distribution with *δ* as the maximum distance between class distributions in standard deviations  modify(d,δ):= generate a modified distribution from *d* with *δ* as the maximum distance between class distributions in standard deviations  classifier():= generate an untrained classifier  clone(c):= create a copy of classifier *c*  accuracy(c,d,n):= accuracy for classifier *c* for distribution *d* over *n* samples  $a_{r,\delta }:=$ the accuracy of the classifier for repeat *r* and distance *δ*  $a_{r,\delta }^{*}:=$ the accuracy of the original classifier for repeat *r* and distance *δ*  **for**
r:=1
**to**
*repeats*
**do**    δ:=0    **while**
δ≤maxdelta
**do**      $d_{1}:=dist\left(\delta \right)$      $d_{2}:=modify\left(d_{1},\delta \right)$      $c_{1}:=classifier\left(\right)$      $c_{1}:=t_{unsupervised}\left(c_{1},d_{1},n_{u}\right)$      $c_{1}:=t_{supervised}\left(c_{1},d_{1},n_{s}\right)$      $c_{2}:=clone\left(c_{1}\right)$      $c_{2}:=t_{unsupervised}\left(c_{2},d_{2},1\right)$      $a_{r,\delta }:=accuracy\left(c_{2},d_{2},n_{a}\right)$      $a_{r,\delta }^{*}:=accuracy\left(c_{1},d_{1},n_{a}\right)$      δ:=δ+deltaincrease    **end**  **end**  $\mu_{i}:=\bar{a_{1,i}\ldots a_{n_{r},i}}$  $\mu_{i}^{*}:=\bar{a_{1,i}^{*}\ldots a_{n_{r},i}^{*}}$  $\left\{\mu_{0},\ldots ,\mu_{maxdelta}\right\}$
▹
Result  $\left\{\mu_{0}^{*},\ldots ,\mu_{maxdelta}^{*}\right\}$
▹
Supervised result for comparison

#### 2.3.4. Impact of Changed Bias Between Classes

Another way in which the modified distribution can differ from the original distribution is the bias between the classes. We expect these variations to have a large negative impact on the performance of QUEST. Large variations in the frequency in which classes occur have a strong impact on the location of quantiles on which QUEST is based.

We have conducted experiments on this effect by running experiments with increasing variation in the distribution of samples over the classes. We expect that a larger variation in the distribution results in a decreased performance, both absolute as well as relative to classifiers trained using fully supervised learning. Algorithm 5 summarises this procedure in pseudocode, and the results of this experiment are shown in Section 3.2.4.

**Algorithm 5:** Procedure used for the simulation of QUEST to evaluate the effect of variations in the bias between classes  dist(σ):= generate an initial random class distribution with *σ* as standard deviation in the fractions with which classes occur  modify(d,σ):= generate a modified distribution from *d* with *σ* as standard deviation in the fractions with which classes occur  classifier():= generate an untrained classifier  clone(c):= create a copy of classifier *c*  accuracy(c,d,n):= accuracy for classifier *c* for distribution *d* over *n* samples  $a_{r,\delta }:=$ the accuracy of the classifier for repeat *r* and distance *δ*  $a_{r,\delta }^{*}:=$ the accuracy of the original classifier for repeat *r* and distance *δ*  **for**
r=1
**to**
*repeats*
**do**    σ:=0    **while**
σ≤maxstd
**do**      $d_{1}:=dist\left(\sigma \right)$      $d_{2}:=modify\left(d_{1},\sigma \right)$      $c_{1}:=classifier\left(\right)$      $c_{1}:=t_{unsupervised}\left(c_{1},d_{1},n_{u}\right)$      $c_{1}:=t_{supervised}\left(c_{1},d_{1},n_{s}\right)$      $c_{2}:=clone\left(c_{1}\right)$      $c_{2}:=t_{unsupervised}\left(c_{2},d_{2},1\right)$      $a_{r,\sigma }:=accuracy\left(c_{2},d_{2},n_{a}\right)$      $a_{r,\sigma }^{*}:=accuracy\left(c_{1},d_{1},n_{a}\right)$      σ:=σ+stdincrease    **end**  **end**  $\mu_{i}:=\bar{a_{1,i}\ldots a_{n_{r},i}}$  $\mu_{i}^{*}:=\bar{a_{1,i}^{*}\ldots a_{n_{r},i}^{*}}$  $\left\{\mu_{0},\ldots ,\mu_{maxstd}\right\}$
▹
Result  $\left\{\mu_{0}^{*},\ldots ,\mu_{maxstd}^{*}\right\}$
▹
Supervised result for comparison

## 3. Results

This section describes the results of the conducted experiments to demonstrate the usability of our proposed algorithm.

### 3.1. Results of Experiments Using Real Life Data

#### 3.1.1. Results of the Feasibility Study Based on Correlation

**Figure 6:**
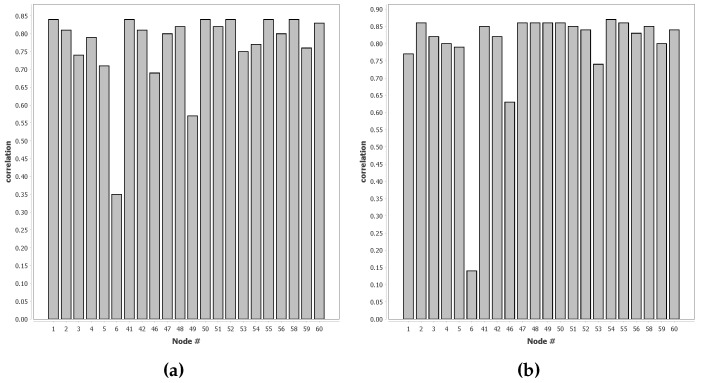
Correlation of class distributions over intervals for the numbered node on the *x*-axis with all other nodes trained using supervised learning. High correlation is an indication that the classifiers work in a similar manner; (**a**) Correlation for AAV (Assault Amphibian Vehicle) vehicles; (**b**) Correlation for DW (Dragon Wagon) vehicles.

In Figure 6, we show, for each baseline classifier for each sensor node, the correlation of the probability distributions over the intervals compared to all the other baseline classifiers, as described in Section 2.2.3. It is clear that the majority of nodes show a high correlation with the other nodes. Apart from a few exceptions, this is a promising result. Node 6 shows a very low correlation, but this, combined with the results from Section 3.1.2, lead us to the conclusion that this node might be malfunctioning.

#### 3.1.2. Performance Compared to Regular Naive Bayes

**Figure 7:**
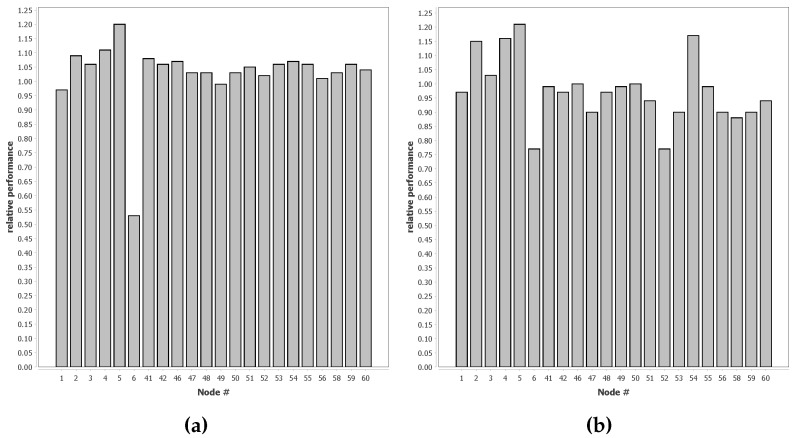
Relative performance for QUEST classifiers compared to fully supervised trained classifiers; (**a**) Relative performance for AAV vehicles; (**b**) Relative performance for DW vehicles.

In Figure 7, we show the results of the experiment described in Section 2.2.4.2. These figures have a column for each sensor. The column for a sensor shows the classification performance of the sensor network if each node starts the online phase with the classifier trained for that node. The shown values are the relative performance compared to the performance of the baseline. The fact that the average relative performance is close to one is an indication that the performance of our approach is very similar to complete supervised learning.

**Figure 8:**
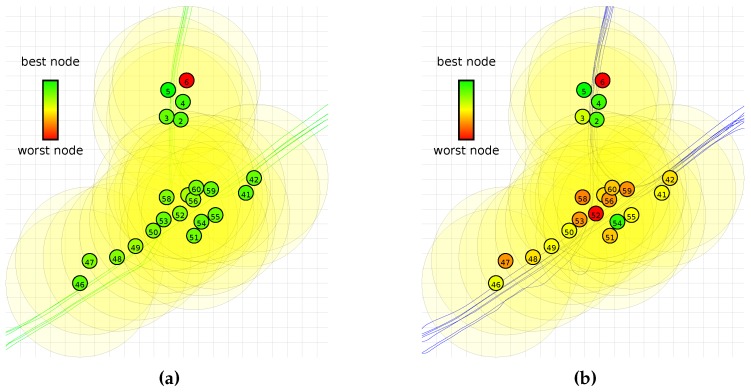
Spatial visualisations of performance of the network when a node is used for the offline phase for the QUEST algorithm. The grid size is 20 m. Colors are assigned on a linear relative scale from red for the worst performing node to green for the best performing node. (**a**) AAV vehicles; (**b**) DW vehicles.

Figure 8 shows a map of the vehicle runs through the target area on which we have visualised the values from Figure 7. In these figures, the best node for that experiment is completely green, and the worst node is completely red. As can be seen, there is no clear clustering of suitable and unsuitable nodes.

### 3.2. Simulation Using Generated Data

#### 3.2.1. Comparing Quantile Estimation Heuristics

**Figure 9:**
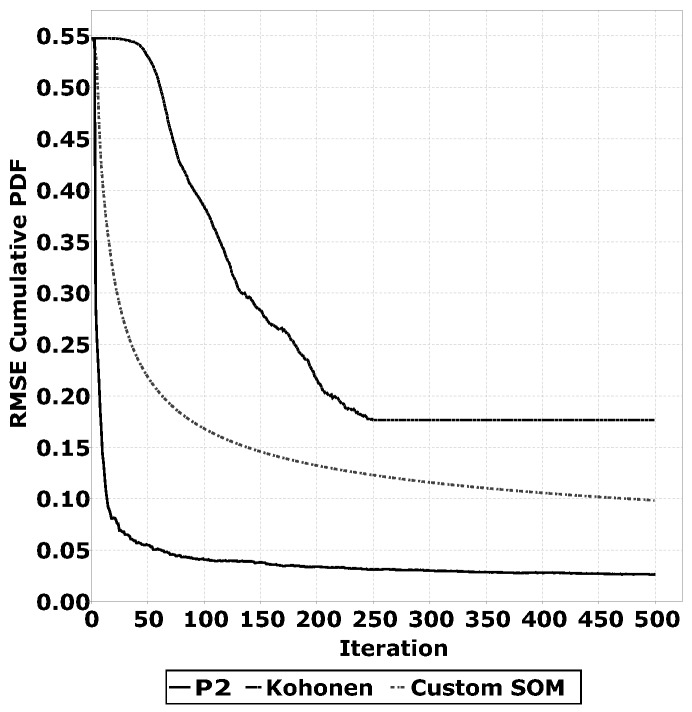
Accuracy of QUEST algorithm when using different quantile estimation heuristics over multiple iterations.

Figure 9 shows the result of the experiment described in Section 2.3.1. As can be seen for all three algorithms ($P^{2}$, Kohonen networks and the custom SOM), the RMSE value converges to a certain accuracy threshold. For the $P^{2}$ algorithm, this threshold is lower than the other two algorithms. Based on these results, we have selected the $P^{2}$ algorithm for the other experiments in this paper.

#### 3.2.2. Accuracy Evolution over Training Iterations

**Figure 10:**
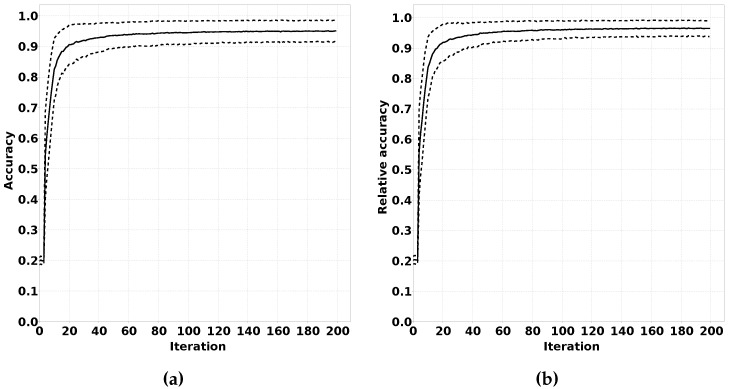
Evolution of accuracy of QUEST classifiers during the online phase with over 200 samples. (**a**) Absolute accuracy; (**b**) Relative accuracy.

In Figure 10, we show the results of the experiment described in Section 2.3.2. The black line shows the average accuracy over 1000 experiments over a number of iterations. The dashed lines show the +σ and −σ lines. Figure 10a shows the absolute accuracy, and Figure 10b shows the accuracy compared to a classifier that was completely trained using supervised learning for the modified distribution. Each repeated experiment was conducted using a different randomly generated pair of distributions. Care was taken to ensure that the distributions of the different classes had sufficient overlap to prevent the classification problem from becoming trivial. In Figure 10b, it is clearly visible that QUEST approaches a near optimal accuracy for the classification problem presented in this experiment.

#### 3.2.3. Impact of the Increased Overlap Between Classes

**Figure 11:**
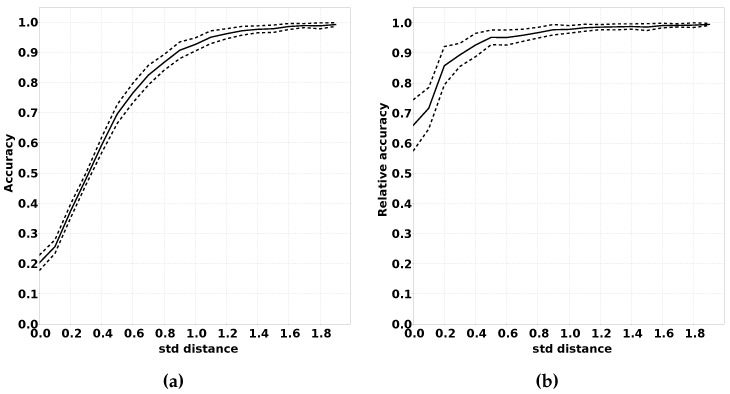
Influence of increasing the number of standard deviations between the mean of data distribution of the output classes. (**a**) Accuracy; (**b**) Accuracy relative to fully supervised trained classifiers.

Figure 11 shows the result of the experiments described in Section 2.3.3. As expected, Figure 11a shows that if classes have a larger overlap, this has a negative impact on classification accuracy. In Figure 11b, we can see that this effect is strongly reduced when we compare the accuracy to a classifier that was trained using complete supervised training. The explanation for this is that it is more complicated for classification algorithms to distinguish between classes with a higher overlap. Therefore, the large impact on accuracy shown in Figure 11a is mostly due to the increased complexity of the classification problem.

#### 3.2.4. Impact of Changed Bias Between Classes

**Figure 12:**
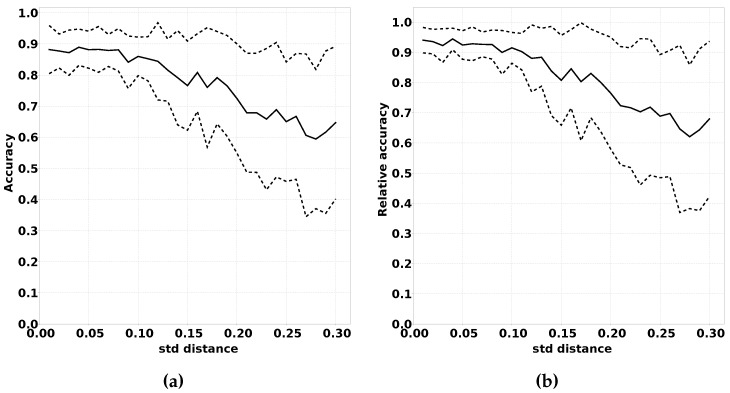
The effect of the variation of the frequency with which classes occur between the offline phase and the online phase. (**a**) Accuracy; (**b**) Relative accuracy.

Figure 12 shows the expected decrease in performance described in Section 2.3.4. Both the absolute values shown in Figure 12a as the relative values shown in Figure 12b show that a larger variation in the bias between classes has a negative impact on performance.

## 4. Conclusions

The proof that the principle behind the QUEST algorithm works is clearly demonstrated in Figure 10. This figure shows that a QUEST classifier can be modified for a new classification problem using unsupervised learning. In Section 3.1.2, we demonstrate that QUEST also works on a real-life classification problem. QUEST is clearly able to adapt a general classifier trained for one situation to a new situation resulting from different circumstances. Figure 10b shows that the performance can closely match the optimal level in a simulated environment. Furthermore, Figure 7 shows that QUEST classifiers can closely match the performance of supervised learning in real-life classification problems. In 90% of the tests shown in Figure 7, the decrease in performance is less than 10% when compared to full supervised learning. For some nodes, the network performance is even better compared to the case that each node uses a classifier resulting from an offline phase on its own local supervised training data.

There is some reason for caution, however, as Figure 7 shows that some nodes do not provide suitable data for the offline phase. The exact reason why some nodes work well and others do not is not directly clear from the results. Possible causes could be that nodes are deployed on a location where the distinction between classes in less clear. Another option is that node 6 malfunctions, either by providing erroneous sensor values or timestamps.

Figure 8 does not show a clear spatial relation for the nodes that work well and the ones that do not. In practice, however, this is not necessarily a problem. The offline phase can be run in a controlled environment, allowing the classifier for the offline phase to be better trained than the ones used in this dataset. If node 6 is indeed malfunctioning and removed from the results, more than 95% of the tests have less than a 10% performance penalty.

Another reason for caution is the effect changed bias between output classes has on the performance of the classifiers. Figure 12a,b demonstrates the negative impact of a large change in class bias. It is our belief, however, that, for the majority of applications, these effects will be minimal. For fire detection, for example, the classification “fire detected” will probably remain a rare classification for which the sensor readings are positioned in the highest and lowest quantiles.

The results presented in this work demonstrate that the application of QUEST enables the deployment of a WSN that requires no on-site communication for training and minimal transmission of data during operation. As such, we believe that QUEST is a valuable contribution to the field of Wireless Sensor Networks.

## 5. Future Work

The results of this research show the potential of the proposed approach and demonstrate the value of this direction of research. There is definitely space for future research. In this section, we describe topics that may lead to interesting results.

### 5.1. Suitable Classification Problems

Section 3.1.2 shows that not all nodes are suitable to be used in the offline phase. Although, for this experiment, this was probably the result of a malfunctioning node, it is possible that there are classification problems where our solution will not work. A full investigation of the types of changes that sensor nodes can encounter when deployed on a different location will provide valuable insight on when our approach is suitable and when there might be complications. In such an investigation, the experiments conducted for this paper can be repeated on different data sets for different classification problems. The most suitable classification problems for these experiments are classification problems with events that can be detected by multiple sensor nodes at the same time. For the SITEX02 dataset, the detection of a vehicle in the range of a single sensor node is a local event for that sensor, which complicates collaboration between sensor nodes. For shared events, Naive Bayes by itself can be used for data fusion, without the need for additional fusion schemes.

### 5.2. Peer Training

If the future work described in Section 5.1 results in classification problems that are not suitable for the presented approach, another approach might be of interest. When on-site supervised training of sensor nodes is necessary, it is interesting to investigate if the supervised learning can be performed by sensor nodes that are already deployed and trained. In this way, the knowledge present in a WSN is propagated over multiple “generations”. Only the first generation needs to be trained by a human supervisor. Sensor nodes installed or replaced later can be trained by the already trained sensor nodes. If this approach is feasible, it would significantly reduce the required human maintenance.

### 5.3. Permanent Adaptability

As shown in Figure 10, the performance of QUEST approaches a certain threshold. Part of this result is caused by the fact that the applied unsupervised learning algorithms use decaying learning factors to reduce the learning effect after a growing number of training iterations. This means that, after a large number of training iterations, the quantile estimations will become almost static. An interesting approach could be to impose a certain minimum value on the learning factor to preserve a certain amount of flexibility. For dynamic classification problems that vary due to, for example, seasonal influences, we believe this could result in increased classification accuracy.
